# The 2015 Dutch food-based dietary guidelines

**DOI:** 10.1038/ejcn.2016.52

**Published:** 2016-04-06

**Authors:** D Kromhout, C J K Spaaij, J de Goede, R M Weggemans

**Affiliations:** 1 The Health Council of the Netherlands, The Hague, The Netherlands

## Abstract

The objective of this study was to derive food-based dietary guidelines for the Dutch population. The dietary guidelines are based on 29 systematic reviews of English language meta-analyses in PubMed summarizing randomized controlled trials and prospective cohort studies on nutrients, foods and food patterns and the risk of 10 major chronic diseases: coronary heart disease, stroke, heart failure, diabetes, breast cancer, colorectal cancer, lung cancer, chronic obstructive pulmonary disease, dementia and depression. The committee also selected three causal risk factors for cardiovascular diseases or diabetes: systolic blood pressure, low-density lipoprotein cholesterol and body weight. Findings were categorized as strong or weak evidence, inconsistent effects, too little evidence or effect unlikely for experimental and observational data separately. Next, the committee selected only findings with a strong level of evidence for deriving the guidelines. Convincing evidence was based on strong evidence from the experimental data either or not in combination with strong evidence from prospective cohort studies. Plausible evidence was based on strong evidence from prospective cohort studies only. A general guideline to eat a more plant food-based dietary pattern and limit consumption of animal-based food and 15 specific guidelines have been formulated. There are 10 new guidelines on legumes, nuts, meat, dairy produce, cereal products, fats and oils, tea, coffee and sugar-containing beverages. Three guidelines on vegetables, fruits, fish and alcoholic beverages have been sharpened, and the 2006 guideline on salt stayed the same. A separate guideline has been formulated on nutrient supplements. Completely food-based dietary guidelines can be derived in a systematic and transparent way.

## Introduction

Dietary guidelines are evidence-based integrated messages to reduce the risk of chronic diseases for the general population. They summarize and synthesize knowledge regarding nutrients and foods. The first dietary guidelines for the Dutch population appeared in 1986 and comprised five nutrient-based guidelines.^1^ The guidelines were updated in 2006 and consisted of four nutrient-based and four food-based guidelines.^2^

Nutrient metrics for the prevention of chronic diseases have major limitations; for example, total protein, fat and carbohydrate intake are not related to chronic diseases, and individual nutrients, for example, fatty acids and sodium, have limited effects. Increasing evidence from controlled trials on risk factors and prospective cohort studies shows that specific foods and dietary patterns substantially affect chronic disease risk.^3^ Therefore, the 2015 Dutch dietary guidelines are completely food-based.

## Materials and methods

A multidisciplinary committee of 15 scientists was appointed, who filled out a declaration of interest published on the website of the Health Council (www.gr.nl). First, a methodology document was prepared.^4^ The committee evaluated the peer-reviewed literature on the relationships among nutrients, foods, food patterns and the risk of the 10 major diet-related chronic diseases based on mortality, life-years lost and burden of disease in The Netherlands. The diseases are as follows: coronary heart disease (CHD), stroke, heart failure, type 2 diabetes, chronic obstructive pulmonary disease, breast cancer, colorectal cancer, lung cancer, dementia and depression. The committee selected also three risk factors—systolic blood pressure, low-density lipoprotein (LDL)-cholesterol and body weight—that are causally related to at least one of the following chronic diseases: CHD, stroke, heart failure and type 2 diabetes.^4^ These risk factors are not causally related to the other six chronic diseases.

The committee selected prospective cohort studies in which the diet was assessed before the disease was diagnosed, because food intake data are more reliable when estimated before disease occurs. The guidelines are also based on randomized controlled trials (RCTs). Both types of prospective studies have advantages but also disadvantages.^5^ RCTs have the advantage of exclusion of confounding and provide strong evidence for causality but generally include selected populations with short follow-up periods. Prospective cohort studies are generally characterized by large populations and long follow-up periods but can never rule out residual confounding.

The committee limited the literature search to studies on adults and children from the age of 2 years onwards and did not include studies on pregnant or lactating women. The literature search of the committee was primarily restricted to pooled analyses, meta-analyses and systematic reviews published in peer-reviewed journals. Literature searches for the background documents covered publications up to July 2014 in PubMed. The committee only included the results of pooled analyses or meta-analyses published thereafter if they either were the first one or reported deviant conclusions from previous meta-analyses.

The committee evaluated the state-of-the-art of science on nutrition and chronic diseases described in 29 background documents. The formulation of the guidelines is only based on conclusions with strong evidence, but it differs for RCTs and cohorts studies. The committee used the word 'effect' for RCTs on dietary factors and causal risk factors or chronic disease incidence and 'association' for cohort studies of dietary factors and chronic disease incidence. The level of evidence depended on the availability and quality of the research, the strength of the associations and the presence of heterogeneity in meta-analysis. Finally, the health council provided the opportunity to comment on drafts of the background documents by public consultation. Comments of stakeholders and responses of the committee have been published on the website of the Council (www.gr.nl) in Dutch.

For the integration of the results into guidelines, foods and nutrients were classified with the food education message to the consumer in mind. The committee composed the following groups: vegetables and fruit, protein-rich foods, carbohydrate and fibre-rich foods, fat-rich foods and fish, beverages, salt, food patterns and nutrient supplements. Next, the committee described for each food and the associated nutrient(s) the results from RCTs and/or cohort studies with strong evidence. The conclusion(s) on which the guidelines were based are described in Supplementary Tables 1–17 in Supplementary Appendix 2. If available, information on associations of the dietary factor with other chronic diseases is also described. In the tables, the quantitative results are specified as comparisons between high and low intake levels or in terms of dose–response relations. The relative risks are rounded off to 5 or 10% in order to avoid seeming accuracy.

Judgements were made on the totality of evidence of the selected findings. If the results of cohort studies on chronic diseases and at least one individual RCT with disease as end point were consistent, the committee regarded the evidence as convincing. The committee called the evidence also convincing if the results of the cohort studies and RCTs with a causal risk factor were consistent. Finally, a significant effect on a causal risk factor was also called convincing. If only results of cohort studies were available, the committee judged the association plausible. Only in the case of convincing evidence from both cohort studies and RCTs the guideline is quantified by means of the consumption levels observed in cohort studies. Consumption levels are much higher in RCTs because their objective is to show a causal relation. If the evidence concerns the replacement of a food by another one, the guideline is formulated in terms of replacement.

## Results

Most data relate to effects observed in adults, whereas data on children aged 2 years and older are scarce.The committee provides guidelines for five food groups, two groups of beverages and one group of food patterns. In addition, guidelines on salt and nutrient supplements are derived. First, the conclusions from the background documents are described that are relevant for the guideline. Thereafter, the committee formulates the guideline.

### Vegetables and fruit

Vegetables and fruit are defined based on their nutritional value, taste and culinary application of plant foods. Although cucumber, tomatoes and red pepper are fruits in the botanical classification, they are classified as vegetables. In addition, green peas, sugar peas, broad beans and butter beans are classified as vegetables. Vegetable juices are not included in the definition of vegetables. In the different studies, fruit includes fresh fruit but also dried and canned fruit and sometimes fruit juice.^6^ In addition, the results of fruit fibre are evaluated including pectin.

The committee concludes that the consumption of vegetables and fruit convincingly reduces the risk of CHD and stroke because the results from RCTs and cohort studies support each other. Vegetables and fruit reduced blood pressure^7, 8, 9, 10, 11, 12^ and were associated with a lower risk of CHD^13, 14, 15^ and stroke.^16^ The effect of pectin in fruit on LDL-cholesterol also supported the causal relation of fruit consumption with CHD risk.^17^ Green leafy vegetables and fruit were also associated with lower diabetes risk,^6, 18^ vegetables and fruits with lower colorectal cancer risk.^19^ Fruit consumption was associated with a lower risk of lung cancer.^20, 21, 22^ Main conclusions supporting the guideline on vegetables and fruit are shown in Supplementary Table 1 in Supplementary Appendix 2.

#### Guideline

Eat at least 200 g of vegetables and at least 200 g of fruit daily.

### Protein-rich products

#### Meat

The major types of meat discriminated in the studies are red, processed and white meat. Red meat is derived from mammals such as cows, calves, pigs, goats, sheep and horses. Processed meat is smoked or salted for conservation purposes or when preservatives such as nitrate or nitrite are added. Processed meat comprises cured meat products such as ham, bacon, sausages and ready-to-eat minced meat. White meat is derived from chickens, turkeys, ducks, geese and domesticated rabbits.^23^

The committee concludes that it is plausible that the consumption of red meat and processed meat is associated with a higher risk of stroke,^24, 25^ diabetes,^26, 27, 28, 29^ colorectal^30, 31, 32, 33, 34, 35^ and lung cancer^36, 37^ (Supplementary Table 2 in Supplementary Appendix 2). The associations were stronger for processed meat than for red meat. There is insufficient evidence for associations of white meat with chronic diseases.^29, 32, 35, 36, 38, 39^

#### Guideline

Limit the consumption of red meat, particularly processed meat.

#### Dairy

Dairy includes, among others milk, yogurt and cheese. Butter is excluded from the definition of dairy, as it is included in the food group fats and oils.

The committee concludes that it is plausible that the consumption of dairy and milk is associated with a lower risk of colorectal cancer^40, 41^ and the consumption of yogurt with a lower risk of diabetes^42^ (Supplementary Table 3 in Supplementary Appendix 2). The conclusion about colorectal cancer is supported by the finding that the intake of calcium from supplements was associated with a lower risk of this disease.^31, 43, 44^ The calcium intake from supplements was approximately about half the amount from dairy.^45^ A distinction between the effects of low-fat and high-fat dairy produce was not possible, because of insufficient evidence.

#### Guideline

Take a few portions of dairy produce daily, including milk or yogurt.

#### Eggs

Eggs are not only a source of protein but also a source of dietary cholesterol (200 mg of cholesterol per egg). In the Dutch food pattern, other important sources of cholesterol are meat and milk products.^45^ In 2006, the Health Council decided not to formulate a guideline for eggs or dietary cholesterol,^2^ and the present committee concludes that more recent evidence does not warrant changing this. The available evidence shows that the intake of 100 mg of cholesterol from eggs increased LDL-cholesterol by 0.05 mmol/l.^46, 47^ These results are from controlled experiments in which the consumption of eggs was considerably larger than habitual. In cohort studies, there is no association between eggs and CHD risk.^48, 49^ It is plausible that the consumption of seven eggs per week and a high intake of dietary cholesterol are associated with a higher risk of diabetes.^49^ However, the intake of cholesterol-rich foods is still unaltered and low in the Dutch population.^45^ The 2006 guidelines stated that a more than average consumption of cholesterol-rich foods is not desirable.^2^ The committee subscribes this and recommends monitoring the consumption.

#### Legumes

Legumes are defined as (soya) beans, lentils, chick peas and split peas. Green peas, sugar peas, broad beans and butter beans belong to the green vegetables and are reviewed together with vegetables and fruit. Most studies included peanuts as nuts.

The committee concludes that the consumption of legumes convincingly reduces LDL-cholesterol^50, 51^(Supplementary Table 4 in Supplementary Appendix 2), a causal risk factor of CHD. However, there are insufficient data from cohort studies on the association between legumes and CHD^52^ to quantify the guideline.

#### Guideline

Eat legumes weekly.

#### Nuts

The committee defines nuts similarly to most researchers and consumers, and it does not use the botanical classification. Among the most frequently consumed ones are walnuts, almonds, hazel nuts, cashew nuts, pistachios, macadamia nuts, Brazil nuts and pecans. Peanuts are also included.

The committee concludes that the consumption of nuts convincingly reduces CHD risk (Supplementary Table 5 in Supplementary Appendix 2). Consumption of nuts reduced LDL-cholesterol in RCTs^53, 54, 55^ and was associated with a lower risk of CHD in cohort studies.^52, 56^ In addition, the PREDIMED-RCT showed that the consumption of 30 grams of nuts per day reduced the incidence of cardiovascular diseases in high-risk patients.^57^

#### Guideline

Eat at least 15 g of unsalted nuts daily.

### Carbohydrate- and fibre-rich products

Cereals consist, among others, of wheat, rice, oats, rye and barley. Examples of cereal products are bread, cereals, crackers, flour, pasta and so on. In The Netherlands, whole-grain bread must consist of 100% whole-grain flour, but the qualification of whole-grain is not protected for other products. In research on diet and chronic diseases, products are frequently called whole-grain if they consist for at least 25% of whole-grain flour.^58, 59, 60, 61^ Potatoes are also a source of starch and fibre. However, there is a lack of scientific information on their health effects. Dietary fibre is a collection of compounds with various physiological functions. The fibre intake in cohort studies concerns fibres of natural sources—for example, wholemeal bread, legumes, potatoes, vegetables and fruit. β-Glucan is a type of fibre in oats and barley.

The committee concludes that the consumption of whole-grain products convincingly reduces the risk of CHD and dietary fibre reduces the risk of stroke (Supplementary Table 6 in Supplementary Appendix 2). The results of RCTs and cohort studies on whole-grain products and fibre are consistent. Dietary fibre reduced diastolic blood pressure in RCTs^62, 63^ and the risk of CHD^64^ and stroke^65, 66^ in cohort studies. In addition, RCTs showed that oats^67^ and β-glucan^17, 68, 69, 70^ reduced LDL-cholesterol. In cohort studies, a high intake of cereal fibre^64^ and whole-grain products^71, 72, 73, 74^ was associated with a lower risk of CHD. A high intake of dietary and cereal fibre and whole-grain products was also related to a lower risk of diabetes^75, 76, 77^ and colorectal cancer.^78, 79, 80, 81^

#### Guidelines

Eat at least 90 g of brown bread, wholemeal bread or other whole-grain products daily. Replace refined cereal products by whole-grain products.

### Fat-rich products and fish

#### Fats and oils

Fat-rich products—for example, butter, margarine and oil—contain a combination of various fatty acids. Until the 1990s, the Dutch diet contained a large amount of *trans-*fatty acids, but nowadays the intake is below 1% of energy. This major change was brought about after it became clear that *trans-*fatty acids increase the risk of CHD. *Trans*-fatty acids are still present in bakery, meat and dairy products. Butter contains more saturated fatty acids than soft margarines and liquid oils. Olive oil contains mostly cis-monounsaturated fatty acids. Sunflower oil contains a lot of *cis*-unsaturated fatty acids, of which two-thirds are polyunsaturated fatty acids. Vegetable fats and oils contain generally a small amount of saturated and a large amount of unsaturated fatty acids. Exceptions are palm oil, coconut fat and cocoa butter, which contain a lot of saturated fatty acids.

The committee concludes that foods rich in *cis*-unsaturated fatty acids, such as soft margarines or vegetable oils, convincingly reduce the risk of CHD compared with foods rich in saturated fatty acids such as butter and hard margarines (Supplementary Table 7 in Supplementary Appendix 2). The results of the RCTs showed a reduction in LDL-cholesterol when butter was replaced by soft margarine^82^ and when saturated fatty acids was replaced by unsaturated fatty acids.^83^ Replacement of saturated fatty acids by polyunsaturated fatty acids reduced the risk of CHD.^84, 85, 86, 87, 88^ This is confirmed in cohort studies.^89^ The PREDIMED-RCT showed that 50 ml of olive oil per day reduced the risk of cardiovascular diseases in high-risk patients.^57^

*Trans*-fatty acids increase the risk of CHD convincingly. RCTs showed that replacement of 1% of energy from *trans-*fatty acids with unsaturated fatty acids reduced LDL-cholesterol by 0.04 mmol/l.^83, 90^ *Trans-*fatty acid intake was associated with a higher risk of CHD in cohort studies.^88, 91^

The current intake of *trans-*fatty acids is below 1% of energy.^45^ The Committee concludes that for such a low level of *trans*-fatty acids a guideline is not needed, but monitoring of the intake is required because of the negative health effects of a higher intake of *trans-*fatty acids.

#### Guideline

Replace butter, hard margarines and cooking fats with soft margarines, liquid cooking fats and vegetable oils.

#### Fish and fish fatty acids

Fish is an important source of the very long-chain polyunsaturated fatty acids eicosapentaenoic acid and docosahexaenoic acid and essential nutrients such as vitamin D, iodine and selenium. Examples of oily fish are herring, salmon and mackerel, and examples of lean fish are cod, plaice and coal-fish.

The committee concludes that eating fish convincingly reduces the risk of fatal CHD (Supplementary Table 8 in Supplementary Appendix 2). The fish fatty acids eicosapentaenoic acid and docosahexaenoic acid reduced in RCTs the risk of fatal CHD in cardiac patients and high-risk groups,^92, 93, 94, 95, 96, 97^ and the consumption of one serving of fish per week was associated with a lower risk of fatal CHD in cohort studies.^98, 99^ In addition, one RCT showed that two servings of oily fish per week reduced the risk of fatal CHD in cardiac patients.^100^ In addition, cohort studies showed that the consumption of one serving per week was associated with a lower risk of stroke.^101, 102, 103^ At a consumption level of one serving per week, there is no evidence for toxicological risks if a variety of different types of fish are eaten.^104^

#### Guideline

Eat one serving of fish, preferably oily fish weekly.

### Beverages

#### Tea

Tea is defined as black and green tea. Green tea is derived from the tea plant but the leaves have not undergone an oxidation process in contrast to black tea. Herb teas and, for example, rooibos tea were not reviewed.

The committee concludes that the consumption of tea convincingly reduces the risk of stroke (Supplementary Table 9 in Supplementary Appendix 2). RCTs showed that three cups of green tea^105, 106^ and five cups of black tea^107^ reduced blood pressure, and cohort studies showed that the consumption of tea was associated with a lower risk of stroke.^108^ The consumption of black tea and green tea was also associated with a lower risk of diabetes.^109^

#### Guideline

Drink three cups of tea daily.

#### Coffee

For coffee, it is relevant to know in which way it is prepared—with a filter or not—because the filter can take away the cholesterol-elevating compounds kahweol and cafestol.^110^ Coffee pads, dissolved coffee and machine coffee based on liquid coffee concentrate are examples of filtered coffee. Examples of unfiltered coffee are boiled coffee, caffetiere coffee, Greek coffee and Turkish coffee. Espresso and some types of machine coffee can be filtered or unfiltered depending on the machine, type and amount of coffee and the type of filter used.^111^

The committee concludes that in RCTs unfiltered coffee convincingly increases LDL-cholesterol^112^ (Supplementary Table 10 in Supplementary Appendix 2), a causal risk factor of CHD. Coffee was associated with a lower risk of CHD, stroke and diabetes in recently carried out cohort studies.^113^ These studies concern mostly filtered coffee.

#### Guideline

Replace unfiltered coffee by filtered coffee.

#### Sugar-containing beverages

Sugar-containing beverages such as drinks with added sugar and fruit juice have similar sugar content. Beverages with added sugar are cold drinks with extra sucrose, fructose or glucose. Examples of sugar-containing beverages are fruit juice, soda, ice tea, vitaminated water, sport beverages and sweetened dairy drinks.

The committee concludes that the consumption of beverages with added sugar convincingly increases the risk of diabetes (Supplementary Table 11 in Supplementary Appendix 2). RCTs showed that these beverages increased body weight,^114^ and cohort studies pointed to an association with diabetes.^115, 116^ These results can also be applied to other beverages such as fruit juice and sweetened dairy drinks. Good alternatives for sugar-containing beverages are tea and filtered coffee without sugar and water.

#### Guideline

Minimize the consumption of sugar-containing beverages.

### Alcoholic beverages

In The Netherlands, a standard glass of alcoholic beverages amounts to ~10 g of alcohol, equivalent to 13 ml of alcohol. That amount of alcohol is present in 250 ml of beer (5% alcohol), 100 ml of wine (12% alcohol) and 35 ml of spirits (35% alcohol).

The committee concludes that a high intake of alcohol convincingly increases the risk of stroke and that binge drinking (60 g or more at one occasion) increases the risk of CHD (see Supplementary Table 12 in Supplementary Appendix 2). RCTs showed that decreasing a high level of alcohol intake reduced blood pressure.^117^ Cohort studies showed that a high (30–60 g per day) compared with a moderate intake of alcohol (1–15 g per day) was associated with a higher risk of stroke,^118^ and binge drinking was associated with a higher risk of CHD.^119^ In addition, a high intake of alcohol was associated with a higher risk of breast cancer^120, 121, 122^ and colorectal cancer,^123, 124, 125^ and a high consumption of beer and spirits was associated with a higher risk of lung cancer.^126, 127^

Low levels of alcohol intake (<15 g per day) were associated with a lower risk of cardiovascular diseases,^118, 128^ diabetes^129, 130, 131^ and dementia^132^ and with a higher risk of breast cancer^120, 121, 122^ as compared with (almost) no alcohol intake. A low level of beer among men and a low level of spirits among women were associated with a higher risk of diabetes,^130^ and a low level of beer and wine was associated with a lower risk of lung cancer.^126, 127^ The associations of low alcohol intake with the risk of chronic disease are shown in Supplementary Table 13 and with the risk of all-cause mortality in Supplementary Table 14 in Supplementary Appendix 2.

A low level of beer consumption was associated with a higher risk of all-cause mortality.^133^ A low level of wine consumption was associated with a lower risk of all-cause mortality.^133^ One glass of alcohol every two days was associated with a lower risk of all-cause mortality.^133, 134^

#### Guideline

Do not drink alcohol or do not drink more than one glass daily.

### Salt

Salt (sodium chloride) is present in many foods and is added to foods. Of the total salt content of the diet, ~20% is added in the kitchen or at the table and about 80% in foods as purchased. Foods often containing much salt are bread, cheese, sausages, hearty snacks and ready-to-eat products. One gram of sodium is equivalent to ~2.5 g of salt.

The committee concludes that, in a large number of RCTs, lowering sodium intake reduces blood pressure^135, 136, 137^ (see Supplementary Table 15 in Supplementary Appendix 2), a causal risk factor of cardiovascular diseases. The protective effect of a low intake of sodium was stronger in people with hypertension than in those with normotension.^135, 136, 137^ The guideline could not be quantified because of insufficient data from high-quality cohort studies on sodium intake and cardiovascular risk. Therefore, the committee decided to stay with the 2006 Dutch guideline that recommended limiting the intake of salt to 6 g per day.

#### Guideline

Limit salt intake to 6 g daily.

### Nutrient supplements

Nutrient supplements are vitamins and/or minerals that are taken in addition to the usually consumed diet. These supplements are available in the form of powders, pills, drops and effervescent tablets. In RCTs, the supplements are generally taken in high dosages that cannot be obtained from the usual diet.^138^

The committee concludes that in RCTs β-carotene supplements convincingly increase the risk of lung cancer in smokers and asbestos workers.^139, 140^ Supplements with vitamin D and calcium convincingly reduce the risk of fractures in the elderly and postmenopausal women.^141^ These effects are shown in Supplementary Table 17 in Supplementary Appendix 2. The committee also concludes that there is insufficient evidence for an effect of vitamin C supplements on cardiovascular risk. Supplementation with vitamin C reduced blood pressure,^142^ but one RCT showed that the effect of 500 mg of vitamin C per day did not affect cardiovascular risk.^143^

#### Guideline

Nutrient supplements are not needed, except for specific groups for which supplementation applies— for example, groups that need extra vitamin D, folic acid or vitamin B12.

### Dietary patterns

Dietary patterns are defined on the basis of the amounts, ratios, variation and the combination of different foods and beverages and the frequency in which they are used.^144^ There are many food-based recommended dietary patterns. Examples are the traditional Mediterranean diet, the new Nordic diet and the American Dietary Approaches to Stop Hypertension diet. These patterns are characterized by basic foods that may differ in quantities. They score high on vegetables, fruit, whole-grain products, nuts, legumes, oils rich in *cis*-unsaturated fatty acids, low-fat dairy, poultry and fish, and score low on red and processed meat, high-fat dairy, hard fats, salt, sugar-sweetened beverages and moderate on alcohol. The patterns are characterised by more plant foods and less animal foods. Vegetarian patterns are characterised by the absence of meat and sometimes also other animal products.

The committee concludes that recommended dietary patterns convincingly reduce the risk of CHD and stroke (see Supplementary Table 17 in Supplementary Appendix 2). RCTs showed that such dietary patterns reduced blood pressure^145, 146, 147, 148^ and cohort studies were associated with a lower risk of CHD and stroke.^144, 149, 150^ RCTs also showed that vegetarian patterns reduced blood pressure^151^ and that cohort studies were associated with a lower risk of CHD.^152, 153^ The different recommended dietary patterns were also associated with a lower risk of diabetes,^154, 155, 156^ colorectal cancer^157^ and death from all causes^158^ in cohort studies. In high-risk patients, the PREDIMED-RCT showed a protective effect on cardiovascular risk of a Mediterranean-style diet with extra-virgin olive oil (50 ml per day) or an additional amount of nuts (30 g per day) compared with a pattern with less fat.^57^

#### Guideline

Follow a dietary pattern that involves eating more plant-based and less animal-based food, as recommended in the guidelines.

## Discussion

The guidelines provide information about which foods and dietary patterns result in health gain based on state-of-the-art science. The committee judged the underpinning of most guidelines as ‘convincing'; only the underpinning of the guidelines on meat and dairy is ‘plausible'.

The average Dutch consumption pattern already meets the guideline on dairy, but with the other guidelines substantial health gain can be obtained.^45^ However, the maximum health gain of all guidelines together cannot be quantified. Most relative risks in the tables are in the order of magnitude of 10–20% and are small. Because of the correlations among food groups, the effects are not additive. However, the results of the PREDIMED-RCT suggest that the more the guidelines are adhered to, the greater the health gain compared with the findings of the cohort studies on dietary patterns.^57^

The guidelines propagate a shift into the direction of plant foods. It has convincingly been shown that this will result in health gain at the population level. An example is the doubling of the vegetable and fruit consumption compared with the average current consumption. In addition, replacement of refined cereal products with wholemeal bread or other whole-grain products has positive health effects. Health gain can also be obtained by eating more legumes and nuts. Only 10% of the Dutch population already eat small amounts of these products; about half of the population does not eat legumes and nuts or eats only very little.

The consumption of animal products also needs adjustment. Although the scientific data are not as solid as those for plant products, it is plausible that moderation of meat consumption is good for health. The consumption of both processed meat and red meat (especially for men) is on a level that is associated with a higher risk of chronic diseases. In contrast, it is favourable to eat more fish. As only half of the Dutch population eats fish twice or three times a month, an increase to one serving a week is beneficial for health.

In addition, a shift in the consumption of beverages is desirable. Health gain is obtained if sugar-containing beverages in children (average Dutch consumption three quarters of a litre per day) and adults (a quarter to a third litre) are replaced by water or by tea and filtered coffee without sugar. People who drink alcohol should limit the amount to one glass per day and avoid binge drinking.

Health gain can also be expected from limiting salt intake. This can be realized by avoiding processed foods and by not adding salt during cooking or at the table. Furthermore, it is favourable to replace butter, hard margarines, cooking fats by soft margarines, liquid cooking fats and vegetable oils. For special population groups, it is important to use extra vitamin D. In addition, women with the desire to become pregnant have to take extra folic acid, and vegans need extra vitamin B12. For the general population, it is not needed to take nutrient supplements for the prevention of chronic diseases.

Not all diet-related disorders were reviewed for the guidelines such as constipation and dental caries. Still the guidelines promote the prevention of these disorders: a higher intake of fibre prevents constipation and less (frequent) sugar consumption helps to prevent caries.^2, 159^

The guidelines are intended for use in the prevention of chronic diseases in the general population. Pregnant women, newborns and children up to 2 years old are outside the scope of the guidelines. Most research data relate to effects observed in adults. Although the committee included studies in children aged two and older, the data available for this group are scarce.

General

- Follow a dietary pattern that involves eating more plant-based food and less animal-based food, as recommended in the guidelines.

Higher consumption recommended

- Eat at least 200 g of vegetables and at least 200 g of fruit daily.
- Eat at least 90 g brown bread, wholemeal bread or other whole-grain products daily.
- Eat legumes weekly.
- Eat at least 15 g of unsalted nuts daily.
- Eat one serving of fish, preferably oily fish, weekly.
- Drink three cups of tea daily.

Replacement recommended

- Replace refined cereal products by whole-grain products.
- Replace butter, hard margarines and cooking fats by soft margarines, liquid cooking fats and vegetable oils.
- Replace unfiltered coffee by filtered coffee.

Limitation recommended

- Limit the consumption of red meat, particularly processed meat.
- Minimize the consumption of sugar-containing beverages.
- Do not drink alcohol or no more than one glass daily.
- Limit salt intake to 6 g daily.
- Nutrient supplements are not needed, except for people who belong to a group for which supplementation applies.

Maintenance of current consumption recommended

- Take a few portions of dairy produce daily, including milk or yogurt.

In 2011, the Health Council published an advice in which the Dietary guidelines were reviewed on ecological aspects.^160^ Recently, the findings in this advice were confirmed in various peer-reviewed articles.^161, 162, 163, 164^ The Committee compared the findings in that advice with those of the current guidelines and concluded that complying to a number of guidelines will not only result in health gain but also in a lower ecological burden. Limiting meat consumption is also desirable from an ecological perspective. Generally, a more plant-food- and less animal-food-based dietary pattern is associated with a lower ecological burden. It means that for a high dairy consumption moderation is also advisable. This also holds for fish: compared with the 2006 guideline (two servings of fish a week), the current guideline to eat fish once a week results in a lower ecological burden. For fish consumption, it is recommended to emphasize that the types of fish that are not overfished or are cultivated in an environment-friendly manner are eaten. From an ecological perspective, it is not enough to adhere to the guidelines. To limit the food-related ecological burden, also measures are needed in the production lines.^165^

The guidelines deal primarily with nutrition behaviour of the consumer. In addition, other parties have instruments to favourably influence behaviour and to make the healthy choice the easy choice. The earlier edition of the guidelines and the advice of the Health Council on food logos pointed also into this direction (www.gr.nl).^2, 166^ The food industry has possibilities to promote product development and product adjustment and could focus on smaller portion sizes, better labelling of food products and on changes in the composition of foods. During the Dutch EU chair ship in the first half of 2016, special attention will be devoted to product improvement.

Compared with other recent dietary guidelines, the Dutch guidelines are unique as they are exclusively formulated in terms of foods. In contrast, recent guidelines from USA,^167^ Australia^168^ and the Nordic countries^169^ combined the guidelines on foods with those on nutrients. For example, in the 2015–2020 Dietary Guidelines for Americans, vegetables, fruits, fat-free and low-fat dairy, a variety of protein foods and oils are promoted, whereas the recommendations are to limit the intake of saturated fats and trans fats, added sugars and sodium.^167^ Similarly, the Australian Dietary Guidelines encompass recommendations on specific foods that should be enjoyed every day and recommendations to limit the intake of foods containing saturated fat, added salt, added sugars and alcohol.^168^ The Nordic Nutrition Recommendations are phrased in terms of energy density, micronutrient density, carbohydrate quality and dietary fat quality and contain specific recommendations on limiting processed and red meat and the use of salt. They do also describe desired changes in terms of specific food groups that potentially promote energy balance and health in Nordic countries, which are largely in line with the Dutch guidelines.^169^

The Dutch dietary guidelines describe what is currently known about the constituents of a healthy dietary pattern in order to prevent chronic diseases. The Netherlands Nutrition Centre is currently translating these characteristics into public information on healthy eating. In this process, dietary reference values are also taken into account. The Netherlands Nutrition Centre's advice is expected to appear in spring 2016.
