# Supplementary material for: The 2015 Dutch food-based dietary guidelines
Source: Eur J Clin Nutr. 2016 Apr 6;70(8):869–78. doi: 10.1038/ejcn.2016.52 (PMC5399142; doi:10.1038/ejcn.2016.52)
Supplement: Supplementary Information [file ejcn201652x1.pdf]

The 2015 Dutch food-based dietary guidelines: supplementary information

Appendix 1: explanation of how the Health Council of the Netherlands has handled interests of the committee.

Appendix 2: 17 supplementary tables describing the main quantitative conclusions supporting the guidelines on fruit, vegetables, meat, dairy products, legumes, nuts, cereal products, fat-rich products, fish, beverages, alcohol, salt, supplements, and dietary patterns.

Appendix 1: Members and advisors to the committee

Members of Health Council committees are appointed in a personal capacity because of their special expertise in the matters to be addressed. Nonetheless, it is precisely because of this expertise that they may also have interests. This in itself does not necessarily present an obstacle for membership of a Health Council committee. Transparency regarding possible conflicts of interest is nonetheless important, both for the chairperson and members of a committee and for the President of the Health Council. On being invited to join a committee, members are asked to submit a form detailing the functions they hold and any other material and immaterial interests which could be relevant for the committee's work. It is the responsibility of the President of the Health Council to assess whether the interests indicated constitute grounds for nonappointment. An advisorship will then sometimes make it possible to exploit the expertise of the specialist involved. Advisors cannot, however, vote on the formulation of the committee's conclusions or recommendations and they have no responsibility for the contents of advisory reports. During the inaugural meeting the declarations issued are discussed, so that all members of the committee are aware of each other's possible interests.

Composition of the Committee Dutch dietary guidelines 2015:

Members: JB, AWH, JAI, DK, HP (until April 2015), JAR, JCS, PV (until June 2015), MV.

Advisors: JMG, JBG, MTEH, RPM, HP (from April 2015 onwards), AMWJS, PV (from June 2015 onwards), MHZ.

Appendix 2: Supplementary tables

Table 1. Main conclusions supporting the guideline on fruit and vegetables

	RCTs		Cohort studies	
Exposure	Blood pressure	LDL-cholesterol	Coronary heart disease	Stroke
Fruit and vegetables	+400 g/d ►-3 mmHg			
Vegetables			200 v. 125 g/d ♦ -10%	Per 200 g/d ♦ -10%
Fruit			250 v. 50 g/d ♦ -10%	Per 200 g/d ♦ -30%
Pectin		+1 g/d ► -0.05 mmol/l		

► represents an effect; ♦ represents an association.

Table 2. Main conclusions supporting the guideline on meat

	Cohort studies			
Exposure	Stroke	Diabetes	Colorectal cancer	Lung cancer
Total red meat	100-120 g/d ♦ +10%	100 g/d ♦ +15%	100 g/d ♦ +10%	100-120 g/d ♦ +20%
Unprocessed read meat	100-120 g/d ♦ +10%	100 g/d ♦ +15%		
Processed meat	50 g/d ♦ +10%	50 g/d ♦ +20%	50 g/d ♦ +15%	

♦ represents an association.

Table 3 Main conclusions supporting the guideline on dairy products

	Cohort studies	
Exposure	Diabetes	Colorectal cancer
Total dairy products		400 g/d ♦ -15%
Milk		Per 200 g/d ♦ -10%
Yoghurt	≥60 g/d v. <10 g/d ♦ -15%	
Calcium supplements[a]		300 mg/d ♦ -10%

♦ represents an association.

a) The associations observed in cohort studies regarding calcium supplements are reported here because the quantities involved are consistent with normal dairy product consumption. Hence, the findings regarding such supplements are relevant in relation to dairy products. In the Netherlands, 58 per cent of calcium intake derives from dairy products.[45]

Table 4 Main conclusions supporting the guideline on legumes

	RCTs
Exposure	LDL-cholesterol
Legumes	130 g/d ► -0.20 mmol/l
Soya protein v. casein	40 g/d ► -0.15 mmol/l

► represents an effect.

Table 5 Main conclusions supporting the guideline on nuts

	RCTs	Cohort studies
Exposure	LDL-cholesterol	Coronary heart disease
Nuts	35 g/d[a] ► -0.15 mmol/l	15 g/d ♦ -20%

► represents an effect; ♦ represents an association.

a Nuts or linseed.

Table 6 Main conclusions supporting the guideline on whole-grain products

	RCTs		Cohort studies	
Exposure	Blood pressure	LDL-cholesterol	Coronary heart disease	Stroke
Whole-grain products			90 g/d ♦ -25%	
Oat		30-60 g/d		

products		► -0.20 mmol/l		
Beta-glucan		+1 g/d ► -0.05 mmol/l		
Cereal fibre			Per 7 g/d ♦ -15%	
Total fibre	+10 g/d ►-1 to -2 mmHg[a]		Per 7 g/d ♦ -10%	High vs low ♦ -15%

► represents an effect; ♦ represents an association.

a) Diastolic blood pressure.

Table 7 Main conclusions supporting the guideline on fat-rich products

	RCTs		Cohort studies
Exposure	LDL-cholesterol	Coronary heart disease	Coronary heart disease
Soft margarine v. butter	10% of energy[a] ► -0.20 mmol/l		
Cis-monounsaturated fatty acids v. saturated fatty acids	1% of energy ► -0.041 mmol/l		
Cis-polyunsaturated fatty acids v. saturated fatty acids	1% of energy ► -0.051 mmol/l	10% of energy ► -15%	5% of energy ♦ -10%

► represents an effect; ♦ represents an association.

a) 10% of energy is 10 per cent of total daily energy intake.

Table 8 Main conclusions supporting the guideline on fish

	RCTs	Cohort studies
Exposure	Fatal coronary heart disease	Fatal coronary heart disease
Fish		≥ 1/wk v. < 1/mth ♦ -15%[a]
Fatty acids from fish	1 g/d ► -10%[b]	

► represents an effect; ♦ represents an association.

a) Eating 1 portion of fish a week is sufficient to obtain the maximum health gain.

b) In cardiac patients and high-risk groups.

Table 9 Main conclusions supporting the guideline on tea

	RCTs	Cohort studies
Exposure	Blood pressure	Stroke
Tea[a]	3-5 cups/d ► -2 mmHg	3-4 cups/d ♦ -10%

► represents an effect; ♦ represents an association.

a) Black tea and green tea.

Table 10 Main conclusions supporting the guideline on coffee

	RCTs
Exposure	LDL-cholesterol
Unfiltered v. filtered coffee	5-6 cups/d ► +0.30 mmol/l

► represents an effect.

Table 11 Main conclusions supporting the guideline on sugar-containing beverages

	RCTs	Cohort studies
Exposure	Body weight	Diabetes
Drinks with added sugar	1 l/d ► +1 kg[a]<br>250-500 ml/d ► +1 kg[b]	Per 330 ml/d ♦ +20%
Sugars from sugar-containing beverages	+17 energy% ► +1 kg[c]	

► represents an effect; ♦ represents an association.

a) In adults, within a month.

b) In children and adolescents; observation concerns an increase in body mass index equating to roughly 1 kilogram of body weight within six to eighteen months.

c) Within 2.5 months.

Table 12 Main conclusions concerning alcohol consumption of more than 15 grams a day, relative to no alcohol consumption

	RCTs	Cohort studies	
Exposure	Blood pressure	Coronary heart disease	Stroke
Alcohol	Per -10% ► -1.0 mmHg		≥30 g/d ♦ +35%[a]
Binge drinking		♦ +45%[b]	

► represents an effect; ♦ represents an association.

a) Relate to the risk relative to people whose alcohol consumption was more than 0, but less than 15 grams a day.

b) Different definitions of binge drinking were used in the cohort studies. The committee considers binge drinking to be 60 grams of alcohol or more per occasion.

Table 13 Main conclusions concerning moderate alcohol consumption (1 to 15 grams a day), relative to no alcohol consumption

	Cohort studies				
Exposure	Cardiovascular disease	Diabetes	Breast cancer	Lung cancer	Dementia
Beer[a]		>0 g/d ♦ +15% ♂		>0 to 5 g/d ♀ and 5 to 15 g/d ♂ ♦ -20%	
Wine[a]				>0 to 12 g/d ♦ -25%	
Spirits[a]		>0 to 12 g/d ♦ +10% ♀			
Alcohol	>2 to 15 g/d ♦ -20%	>0 to 24 g/d ♀ and 6 to 48 g/d ♂ ♦ -20%	>5 to 15 g/d ♦ +5%[b]		>0 to 30 g/d ♦ -25%

♦ represents an association.

a) The quantities relate to the amount of alcohol in the drink.

b) Relate to the risk relative to people whose alcohol consumption was more than 0, but less than 5 grams a day.

Table 14 Main conclusions concerning associations with all-cause mortality

Exposure	Men	Women	Men and women
Beer[a]	10-20 g/d ♦ +10%[b] >20 g/d ♦ +40%[b]	3-10 g/d ♦ +15%[b] >10 g/d ♦ +50%[b]	

Wine[a]	>0 to 10 g/d ♦ -30%[c]	>0 to 10 g/d ♦ -20%[c]	
	>40 g/d ♦ +20%[b]	>20 g/d ♦ +15%[b]	
Alcohol			6 g/d ♦ -15%[c]

♦ represents an association.

a) The quantities in this table relate to the amount of alcohol in the drink.

b) Relate to the risk relative to people whose alcohol consumption was more than 0, but less than 3 grams a day.

c) Relate to the risk relative to people who did not consume any alcohol.

Table 15 Main conclusions supporting the guideline on salt

	RCTs
Exposure	Blood pressure
Sodium	-2 g/d ► -2 mmHg[a] and -5 mmHg[b]

► represents an effect.

a) People with normal blood pressure.

b) People with high blood pressure.

Table 16 Main conclusions supporting the guideline on supplements

	RCTs		
Exposure	Lung cancer	Fractures (total)	Hip fractures
Beta-carotene supplement	20-30 mg/d[a] ► +20%		
Vitamin D with calcium supplement		10-20 µg/d vitamin D and 1 g/d calcium ► -10%[b]	10-20 µg/d vitamin D and 1 g/d calcium ► -15%[b]

► represents an effect.

a) In smokers and asbestos workers.

b) Elderly people, particularly post-menopausal women.

Table 17 Main conclusions supporting the guideline on dietary patterns

	RCTs	Cohort studies		
Exposure	Blood pressure	Coronary heart disease	Stroke	All-cause mortality
Recommended dietary patterns	► -5 mmHg	♦ -20%	♦ -20%	♦ -20%
Vegetarian dietary patterns	► -5 mmHg	♦ -25%		

► represents an effect; ♦ represents an association.
